# Association Between Cannabis Use and Healthcare Utilization in Patients With Irritable Bowel Syndrome: A Retrospective Cohort Study

**DOI:** 10.7759/cureus.8008

**Published:** 2020-05-07

**Authors:** Parth Desai, Chimezie Mbachi, Ishaan Vohra, Miguel Salazar, Madhu Mathew, Tejinder Randhawa, Zohaib Haque, Yuchen Wang, Bashar Attar, Isaac Paintsil

**Affiliations:** 1 Internal Medicine, John H. Stroger, Jr. Hospital of Cook County, Chicago, USA; 2 Gastroenterology and Hepatology, Rush University Medical Center, Chicago, USA; 3 Gastroenterology and Hepatology, John H. Stroger, Jr. Hospital of Cook County, Chicago, USA

**Keywords:** irritable bowel syndrome, cannabis, functional bowel disease, health care utilization

## Abstract

Introduction

Irritable bowel syndrome (IBS) is a frequent cause of abdominal pain and altered bowel habits, which is associated with significant healthcare utilization. The effects of the active compound of cannabis, Δ9-tetrahydrocannabinol (THC), on gut motility and tone have been studied in several experimental models. It is unknown whether these effects correlate with improved healthcare utilization among cannabis users. The purpose of this study is to evaluate the impact of cannabis use on inpatient length of stay and resource utilization for patients with a primary discharge diagnosis of IBS.

Methods

Data were extracted from the Healthcare Cost and Utilization Project Nationwide Inpatient Sample database from 2010 to 2014 for all patients with a primary discharge diagnosis of IBS. Cannabis users (n=246) and non-users (n=9147) were directly compared for various clinical outcomes.

Results

Cannabis users were less likely to have the following: upper gastrointestinal endoscopy (17.9% vs. 26.1%; adjusted odds ratio [aOR]: 0.51 [0.36 to 0.73]; p<0.001) and lower gastrointestinal endoscopy (21.1% vs. 28.7%; aOR: 0.54 [0.39 to 0.75]; p<0.001). Additionally, cannabis users had shorter length of stay (2.8 days vs. 3.6 days; p=0.004) and less total charges (US$20,388 vs. US$23,624). There was no difference in the frequency of CT abdomen performed.

Conclusions

Cannabis use may decrease inpatient healthcare utilization in IBS patients. These effects could possibly be through the effect of cannabis on the endocannabinoid system.

## Introduction

Irritable bowel syndrome (IBS) is a frequent cause of abdominal pain and altered bowel habits worldwide. Per the Rome IV criteria, the disorder is characterized by recurrent abdominal pain associated with defecation or changes in stool frequency or form [1]. Patients are subtyped based on predominant symptoms of diarrhea (IBS-D) versus constipation (IBS-C) or may be categorized to have mixed (IBS-M) or unclassified IBS. IBS is estimated to affect 10 to 15% of the worldwide population and is among the most frequent digestive diagnoses in ambulatory care settings in the United States [2,3]. Despite being predominantly treated in outpatient settings, IBS patients with severe symptoms are occasionally admitted to the hospital [4]. Consequently, these hospitalizations contribute approximately 25% to 30% of total health expenditures from the illness [4]. The syndrome is not a significant cause of mortality, yet it is associated with substantial healthcare utilization and reduction in quality of life [4]. The cost of IBS has been estimated to be US$949.8 million (direct) and US$57.5 million (indirect), accounting for more than one billion dollars in economic burden [5]. Health-related quality of life (HRQoL) data suggest physical impairment similar to patients with diabetes and a greater degree of impairment than those with depression and gastroesophageal reflux disease [6].

The exact pathophysiology of IBS remains unclear. Proposed mechanisms include gut motility dysregulation, altered microbiomes, visceral hypersensitivity, and altered brain-gut interaction [1,7]. Other factors include infectious exposures, inflammatory triggering, genetic susceptibility, and psychological states [7,8].

Corresponding with the heterogeneity of the disorder's pathophysiological mechanisms and manifestations, a variety of pharmacological agents are used in the treatment of IBS. Treatments are aimed at an individual's predominant symptoms (e.g. diarrhea vs. constipation) and include antispasmodics, antidiarrheals, and intestinal secretagogues. Cognitive behavioral therapy and antidepressants are also often used in clinical practice to help alter central pain processing related to the illness [1,9].

Cannabis and other cannabinoids have emerged as therapeutics for gastrointestinal disorders with symptoms similar to IBS, including inflammatory bowel disease and chemotherapy-related nausea; thus, they may be potential agents for symptom reduction in IBS [10]. The use of cannabis in the past has been limited by factors such as federal prohibition, cultural attitudes, and lack of randomized controlled trial data [11-13]. However, in the recent two decades, there has been a decline in negative public perceptions regarding its harms [13]. As of March 2020, 33 states and Washington D.C. have passed laws allowing the use of cannabis for medicinal purposes [14].

Cannabis is thought to act in the gastrointestinal tract through Δ9-tetrahydrocannabinol (THC), which binds to G-protein coupled cannabinoid receptors, CB1 and CB2. These alter gut motility and colonic tone by lowering the presynaptic release of excitatory neurotransmitters, primarily acetylcholine and substance P, from myenteric neurons [11,15]. Placebo-controlled studies have shown that the use of dronabinol, a synthetic form of THC, is associated with reduced fasting colonic motility and tone in IBS patients [10].

Despite proven effects on gastrointestinal regulation, it is uncertain whether cannabis use is associated with favorable clinical outcomes and resource utilization in patients with IBS. Our study used the Nationwide Inpatient Sample database to evaluate the impact of cannabis use on inpatient length of stay and resource utilization for patients with a primary discharge diagnosis of IBS.

## Materials and methods

Cohort and variables

This study used a population-based cohort database based on the Healthcare Cost and Utilization Project (HCUP) Nationwide Inpatient Sample (NIS) dataset. We extracted five years of data (calendar years 2010 through 2014). The NIS is a yearly survey of 20% of total admissions from more than 4,000 hospitals across more than 30 U.S. states and the District of Columbia. The NIS has been validated in several studies to provide reliable estimates of disease and co-morbidity prevalence among inpatient admissions in the United States [16].

In this study, we analyzed the inpatient data for a cohort of patients with IBS identified through the following primary diagnosis code: 564.1. Cannabis use was defined by ICD-9-CM (International Classification of Diseases, Ninth Revision, Clinical Modification) codes 304.3, 304.3x, and 305.2x as either mild (non-dependent use) or moderate/severe (dependent use), which has also been used in previous studies [17-19].

For each dataset, we extracted demographic factors (gender, age, race), hospital-level characteristics (hospital size, teaching status [teaching vs. non-teaching], and geographic location [region of the United States and rural vs. urban]), health insurance, and income status. Co-morbidity burden was collected and quantified using the Elixhauser Comorbidity Index [20]. Patients with a concomitant diagnosis of inflammatory bowel disease or with missing variables were excluded from the sample population. Our clinical outcomes were lower gastrointestinal endoscopy (LGIE), upper gastrointestinal endoscopy (UGIE), CT of the abdomen, length of stay, and total charge.

Statistical analysis

Cannabis users were compared directly with non-users using the Student t-test, Wilcoxon rank-sum test, or Kruskal-Wallis test to compare continuous variables as guided by the statistical test for normal distributions. Depending on cell size, we used the chi-square test or Fisher exact test to compare categorical variables.

To evaluate the statistical significance of differences in the aforementioned clinical end-points, we built forward stepwise multivariable logistic regression models to establish adjusted odds ratios (aORs) for cannabis use on the rates of LGIE, UGIE, and CT of the abdomen. The selection criteria for entry into the model was a p-value of <0.2, and for retention in the model, it was a 0.1. All statistical analyses were performed using STATA Version 14.0 (StataCorp., College Station, TX, USA). All p-values were two-tailed; p-values of <0.05 were considered to be statistically significant.

## Results

Cohort characteristic and direct comparison

A total of 9,393 adult patients were admitted with a diagnosis of IBS during the study period, among which 246 (2.6%) were coded as cannabis users. Compared with patients without recognized cannabis use, cannabis users were significantly younger (mean age 34 years vs. 51 years; p<0.001), more likely to be male (37.4% vs. 19.2%; p<0.001), African American (26.6% vs. 11.5%; p<0.001), in the lowest quartile of median household income (34.6% vs. 26.6%; p<0.004), and more likely to use alcohol (8.9% vs. 2.0%; p<0.001). Comparison of hospital characteristics revealed significant differences between users and non-users as cannabis users more likely had Medicaid as their expected primary payer (32.5% vs. 16.6%; p<0.001) and less likely to list private insurance as their expected primary payer (22.0% vs. 35.4%; p<0.001) (Table 1).

A direct comparison of co-morbidity profile between users and non-users showed a significantly lower prevalence of selected disease among cannabis users, including congestive heart failure, diabetes, and hypothyroidism, but a significantly higher rate of concurrent psychiatric diseases (Table 1).

**Table 1:** Descriptive statistics of patients admitted with a primary discharge diagnosis of irritable bowel syndrome n, number; SD, standard deviation ‡p-Values obtained using the Kruskal-Wallis test for continuous values and the chi-square test or Fisher exact test for categorical variables. *Categorical variables presented as frequency. †Continuous variables presented as mean value and standard deviations.

	Cannabis exposed	Non-cannabis exposed	p-Value^‡^
Observations, n	246	9147	
Sex, female	62.6	80.8	<0.001
Race, %^*^			<0.001
Caucasian	60.1	76.6	
Black	26.6	11.5	
Hispanic	10.3	8.6	
Asian or Pacific Islander	0	0.9	
Native American	0.4	0.4	
Other	1.9	2.6	
Age, mean (SD), years^†^	34.3 (11)	50.9 (19)	<0.001
Co-morbidities, %^*^			
AIDS	0.4	0.2	0.571
Alcohol abuse	8.9	2.0	<0.001
Deficiency anemia	11.0	14.9	0.089
Arthritis	2.9	4.8	0.163
Blood loss anemia	0.0	0.8	0.157
Congestive heart failure	1.2	4.4	0.015
Chronic lung disease	17.1	20.2	0.233
Coagulopathy	0.4	2.1	0.062
Depression	27.2	24.6	0.350
Diabetes	5.7	13.8	<0.001
Diabetes with chronic complications	2.9	3.0	0.907
Hypothyroidism	3.3	12.9	<0.001
Hypertension	24.8	42.5	<0.001
Liver	6.5	5.6	0.542
Electrolyte derangement	34.6	36.4	0.544
Metastatic cancer	0.0	0.3	0.360
Neurological disorders	3.7	6.4	0.085
Obesity	7.7	12.4	0.028
Paralysis	1.2	0.7	0.298
Peripheral vascular disease	1.2	3.9	0.031
Psychosis	17.1	9.9	<0.001
Pulmonary circulation disorders	0.0	1.1	0.106
Renal failure	2.4	5.2	0.052
Tumor	0.0	0.7	0.206
Valvular heart disease	2.0	2.9	0.408
Elixhauser index score, %^*^			
0-1	17.9	34.5	<0.001
2-3	54.4	41.5	<0.001
≥4	27.6	24.1	0.197
Hospital bed size, %^*^			0.002
Small	12.2	14.8	
Medium	37.6	27.3	
Large	50.2	58.0	
Hospital location, %^*^			0.036
Rural	6.9	9.6	
Urban non-teaching	38.4	43.7	
Urban teaching	54.7	46.7	
Hospital regions, %^*^			<0.001
Northeast	16.3	19.5	
Midwest	26.4	24.6	
South	31.3	39.9	
West	26.0	16.0	
Expected primary payer, %^*^			<0.001
Medicare	13.8	35.7	
Medicaid	32.5	16.6	
Private	22.0	35.4	
Others	30.6	12.1	
Median household income (in quartiles), %^*^			0.004
Q1	34.6	26.6	
Q2	22.9	25.8	
Q3	28.3	25.7	
Q4	14.2	21.9	

When we evaluated clinical end-points, we found that among cannabis users, there was less LGIE (21.1% vs. 28.7%; p<0.010), less UGIE (17.9% vs. 26.1%; p<0.040), shorter lengths of stay (2.8 days vs. 3.6 days; p=0.004), and less total charges (US$20,388 vs. US$23,624) (Table 2). There was no difference in the frequency of CT of the abdomen performed (Table 2).

**Table 2:** Descriptive statistics of healthcare utilization among patients with a primary diagnosis of irritable bowel syndrome USD, U.S. dollars; CT, computed tomography; LGIE, lower gastrointestinal endoscopy; UGIE, upper gastrointestinal endoscopy ‡p-Value obtained using the Kruskal Wallis test for continuous values and the chi-square test or Fisher exact test for categorical variables. †Continuous variables presented as median. *Categorical variables presented as percentage.

	Cannabis exposed	Non-cannabis exposed	^p-Value‡^
Observations, n	246	9147	
Hospital course^†^			
Median total charge (USD)	20,388	23,624	<0.001
Median length of stay (days)	2.8	3.6	0.004
Investigation, %*			
LGIE	21.1	28.7	0.010
UGIE	17.9	26.1	0.040
CT of the abdomen	2.8	3.1	0.755

Univariate and multivariate logistic regression 

In the multivariable logistic regression analysis, cannabis use remained associated with a reduced prevalence of the following outcomes: UGIE (aOR: 0.51 [0.36 to 0.73]; p<0.001) and (LGIE (aOR: 0.54 [0.39 to 0.75]; p<0.001) (Table 3).

**Table 3:** Univariate and multivariate logistic regression of clinical outcomes OR, odds ratio; CI, confidence interval; LGIE, lower gastrointestinal endoscopy; UGIE, upper gastrointestinal endoscopy *Adjusted for age, gender, race, median income quartile, Elixhauser Comorbidity Index score, comorbidities, and hospital and insurance characteristics.

	Cannabis exposed vs. non-cannabis exposed			
Odds of having	Unadjusted OR (95% CI)	Unadjusted p-value	Adjusted* OR (95% CI)	Adjusted* p-value
LGIE	0.67 (0.49-0.91)	0.010	0.54 (0.39-0.75)	<0.001
UGIE	0.63 (0.46-0.88)	0.006	0.51 (0.36-0.73)	<0.001
CT of the abdomen	0.89 (0.42-1.90)	0.760	0.97 (0.44-2.14)	0.948

## Discussion

Our study is the first nationwide cohort study to evaluate the association between cannabis use and healthcare utilization in patients with IBS. We have found that cannabis use is associated with a lower use of endoscopic procedures, lower length of stay, and lower median total cost of hospitalization. We posit that the lower use of endoscopy in cannabis users - and hence lower cost of hospitalization - may be due to a lower symptomatic burden when compared to non-users [9]. These findings may be attributable to the well-studied effects of cannabis’ active compound, THC, on the endocannabinoid system of the gastrointestinal tract. While cannabis itself has not been well-studied in IBS, several studies have evaluated the effects of dronabinol, a synthetic THC oral agent, on intestinal motility and compliance and on visceral perception in IBS patients and healthy volunteers.

A randomized control trial by Wong et al. in 2011 studied the effect of dronabinol on colonic motility and sensation in patients with IBS. They found that dronabinol was associated with reduced fasting colonic motility index in the proximal left colon and distal left colon. Additionally, it was found that colonic compliance was increased. The effects were most pronounced in those with IBS-D and IBS with alternating symptoms [21]. A follow-up study by the same group in 2012 investigated the effect of dronabinol on colonic transit time in patients with IBS-D. This study found that patients with a specific cannabinoid receptor 1 genotype, rs806378 CT/TT, demonstrated a delay in colonic transit when receiving dronabinol compared with the control group. This effect was not seen in patients with other genotypes studied [22]. This suggests that the effects of THC on colonic motility may depend on an individual’s specific cannabinoid receptor genotype. On the contrary, Klooker et al in 2011 conducted a study to assess the effect of dronabinol on sensitivity to rectal distension in 12 healthy volunteers and 10 IBS patients ( IBS-D, 4 IBS-C, and 1 IBS alternating based on Rome II criteria). They did not find significant differences in visceral perception after rectal distension, with and without sigmoid stimulation, between the dronabinol and placebo groups [23]. This finding was consistent in both healthy volunteers and IBS patients.

As elucidated above, the existing clinical research regarding THC and IBS is mostly limited to its effects on short-term symptoms and physiological parameters. Even so, these studies support a potential therapeutic role of THC containing agents in IBS. While the pharmacokinetic profiles and route of administration of dronabinol and cannabis differ, we believe that data on dronabinol may be cautiously extrapolated to cannabis given that their pharmacological effects are posited to be driven by THC.

Our study adds to the literature on IBS and cannabis by presenting data related to in-hospital resource utilization, which may be related to therapeutic effects of cannabis use. Existing data suggest that healthcare costs associated with IBS are driven by diagnostic testing, invasive procedures, and operations [6]. This is consistent with our findings that cannabis users required less lower and upper gastrointestinal endoscopies, with concomitant lower lengths of hospitalizations and lower total costs of care. This could be explained by cannabis users having less symptomatic presentations, hence requiring fewer investigative modalities and inpatient services.

Our study used data from one of the largest databases of hospitalized patients in the United States. However, our study has limitations. First, the time of diagnosis and severity of illness and the concurrent therapeutic regimens of the studied population could not be ascertained from the dataset. Second, ICD-9 coding standards do not stratify patients with IBS by predominant symptom (e.g. IBS-D, IBS-C, IBS-M, or IBS alternating). This is important as THC’s effects on IBS have been shown to be most pronounced in patients with IBS-D, as discussed above. Third, NIS data are only generalizable to the hospitalized populations in the United States, and outcomes following discharge could not be delineated. Fourth, cannabis use may be underestimated given that data were extracted from coded diagnoses and not from direct interview, which may explain a lower prevalence of cannabis use in our study when compared with previous research [24,25]. Cannabis use may additionally be underreported in clinical settings given its federal prohibition. Furthermore, our study lacks data on methods, routes, and dosing of cannabis. Additionally, side effects of cannabis could not be ascertained from the dataset given reliance on coded diagnoses. Despite the aforementioned limitations, the large nationwide cohort, scientific rationale, and methodological rigor of our study provide a unique addition to the literature on the effect of cannabis use on IBS. Our results should be interpreted cautiously at this time but warrant further validation with prospective randomized controlled trials.

## Conclusions

Our study provides evidence to suggest that cannabis use may decrease healthcare utilization and costs among hospitalized patients with IBS. These findings are likely attributable to the effects of cannabis’ active compound, THC, on gastrointestinal motility and colonic compliance. The role of cannabis in the treatment for IBS has potential for significant impact at the individual and population level given the burden of IBS on individual quality of life and healthcare expenditures.

## Appendices

**Table 4:** ICD-9-CM Codes for identifying irritable bowel syndrome, cannabis use (dependent and non-dependent) ICD-9-CM, International Classification of Diseases, Ninth Revision, Clinical Modification

Variables	ICD-9 codes
Irritable bowel syndrome	564.1
Cannabis abuse	
Dependent	304.3, 304.3x
Non-dependent	305.2x
Procedures	
Lower gastrointestinal endoscopy	45.24, 48.23, 45.23, 45.25, 45.22, 48.24
Upper gastrointestinal endoscopy	42.23, 42.24, 44.13, 44.14, 45.13, 45.14, 45.16
CT of the abdomen and pelvis	88.01
